# Supplementary material for: Are carbon emissions trading and green financial instruments synergistic? -Comprehensive quantitative research based on content analysis
Source: PLoS One. 2024 Mar 7;19(3):e0298601. doi: 10.1371/journal.pone.0298601 (PMC10919601; doi:10.1371/journal.pone.0298601)
Supplement: S1 Data — (ZIP) [file pone.0298601.s001.zip › Data/地级市Z_F工作报告.zip!/╔╜╢1⁄2/╢1⁄2╙¬╩╨/└┤╫╘╣1⁄2╓┌║┼í╛┬φ┐╦╩2╛▌═°í┐_2016.docx]

各位代表：

　　现在，我代表市人民政府向大会报告政府工作，请予审议，并请市政协委员和其他列席会议的同志提出意见。

　　一、“十二五”经济社会发展规划完成情况

　　“十二五”时期是很不寻常、很不平凡的五年，面对复杂形势和严峻挑战，在市委的正确领导下，全市上下坚持以实施黄蓝国家战略为统领，在迎接挑战中砥砺奋进，在抢抓机遇中阔步前行，胜利完成“十二五”规划确定的主要目标任务，改革开放和现代化建设取得新的重大成就。

　　经济综合实力迈上新台阶。生产总值、规模以上工业主营业务收入、公共财政预算收入分别达到3450.6亿元、1.3万亿元、220.1亿元，年均增长10.6%、16.8%、16%，人均GDP、人均财力居全国地级市前列，主营业务收入过百亿企业达到32家，山东百强企业我市占22%。固定资产投资累计完成1.2万亿元，是“十一五”的2.5倍。金融机构各项存款余额3683.5亿元、贷款余额3031.2亿元，分别是2010年的2.4倍和2.6倍。

　　产业结构调整实现新突破。三次产业结构由3.7：72.6：23.7调整为3.4：64.7：31.9。积极推进新型工业化，高新技术产业和装备制造业年均增速高于规模以上工业，高新技术产业比重提高9.7个百分点。服务业增加值年均增长10.6%，比重提高8.2个百分点。实施省级以上科技计划项目367项，新增科技型企业348家，积极创建全国质量强市示范城市，建设了一批重大创新和质监平台。知识产权工作走在全国地级市前列。

　　“三农”工作取得新成效。强农惠农富农政策全面落实，五年市财政支农资金53.3亿元。产业结构不断优化，粮食综合生产能力明显增强，畜牧、渔业、食用菌等主导产业加快发展，肉蛋奶人均占有量居全省首位。被整建制列为国家现代农业示范区，各类农业园区达到190个。产业化水平不断提高，龙头企业发展到631家。新型农业生产经营主体不断壮大，农民专业合作社发展到1817家，家庭农场发展到963家。黄河口农产品品牌竞争力不断提升，24个产品被评为中国和山东名牌产品。农业农村基础设施不断完善，连续开展大规模农田水利基本建设，解决了26.8万农村人口的饮水安全问题，新建改造农村公路2372公里。新型农民学校培训203万人次。

　　城乡区域一体化发展迈出新步伐。常住人口城镇化率达到65.5%，比2010年提高5.5个百分点。加快中心城规划建设，实施了规划展览馆、大剧院、金湖银河等一批重大工程项目，建成区面积达到188.2平方公里，荣获中国人居环境奖。县城和小城镇快速发展，新型城乡社区规范化建设扎实推进。胜利油田改革创新、转型发展取得新成效。市和油田建立联席会议制度，共同建设特色产业园、科技创新园和大学生创业园，共同推进城市建设、环境治理和民生改善，团结共建开创新局面。军地协商解决了一批事关区域发展的重大问题。油地校三方合作共建中国石油大学胜利学院取得显著成效。探索建立了油地军校信访维稳联动机制。各县区、市属开发区竞相发展，广饶县、垦利县进入全国工业百强县。东营港新增码头29个，东营至旅顺滚装航线复航，靠泊能力达到8万吨级，吞吐能力达到5000万吨。广利港主体工程全面开工，疏港道路通车，临港产业区配套不断完善。胜利机场增开优化航线8条。寿平铁路建成通车，黄大铁路开工建设。横跨黄河500千伏输变电工程、胜利电厂三期竣工投用。

　　生态环境建设得到新加强。建成生态林场11处，新增造林面积36万亩，创建为国家园林城市。自然保护区修复湿地30万亩，城市湿地建设顺利推进。开展乡村文明建设专项行动，县乡统筹的环卫运行和经费保障机制实现全覆盖。实施环保重点工程582项，新建改造城乡污水处理厂45座，重点河流水质稳定好转，空气环境质量得到改善。淘汰落后产能项目122个，圆满完成“十二五”节能减排目标任务。

　　改革开放开创新局面。改革工商登记制度，新增各类市场主体6.7万户。财税体制改革不断深化，政府预决算公开、营改增试点扎实推进。金融体系进一步完善，新增开业银行15家。企业改革取得新成效，新增上市企业2家，一批企业在股权交易市场挂牌。农村综合改革继续深化，在全省率先完成农村土地承包经营权确权登记颁证，供销社综合改革试点全面展开。在全省率先启动全国低丘缓坡荒滩未利用地等开发试点。公立医院综合改革纳入国家级联系试点，“三医联动”扎实推进，药品零差率销售在公立医院全面实行。教育领域综合改革不断深化，办学体制、考试招生等改革取得新成效。市县政府机构改革全面完成，事业单位改革扎实推进。累计完成进出口619亿美元，是“十一五”的3倍。橡胶轮胎展、石油装备展成为对外开放的重要平台。企业“走出去”步伐加快。对口支援任务圆满完成。

　　社会民生建设达到新水平。每年实施一批民生实事，五年市财政对民生投入345.4亿元。城乡居民收入分别达到38735元、13887元，年均增长10.2%、13.3%。城镇累计新增就业23万人，农村劳动力转移就业21.6万人。社会保障标准逐年提高，在全省率先整合城乡居民基本医疗保险、基本养老保险制度，建立城乡居民大病保险制度、临时救助制度和城镇职工长期医疗护理保险制度。建设各类保障性住房和棚户区改造安置房9.8万套。新增劳动力平均受教育年限达到14.5年，提高1年。人均预期寿命提高0.9岁，达到78.5岁。获得第三批国家公共文化服务体系示范区创建资格，黄河口国际马拉松赛成为国际田联“银标赛事”。精神文明建设不断加强，成功创建为全国文明城市。“六五”普法全面完成，基层民主进一步扩大。平安东营建设深入推进，社会治安综合治理和信访工作不断加强，城市公共安全专项行动扎实开展，食品药品安全监管力度持续加大，应急管理体系更加完善，社会秩序保持稳定。国防动员、人民防空、民兵预备役建设、双拥共建和优抚安置工作不断加强，军政军民团结更加巩固。妇女儿童、慈善、残疾人、老龄事业持续发展，气象、防震减灾、民族宗教、外事侨务、对台工作成效明显。

　　政府自身建设取得新进展。党的群众路线教育实践活动和“三严三实”专题教育取得扎实成效。严格落实中央八项规定精神，违规公务用车、超标办公用房等问题得到有效整治。认真执行市人大及其常委会的决议、决定，自觉接受人大、政协、群众和舆论监督。行政权力清单、行政审批事项目录清单、政府部门责任清单公布实施，市级政务服务平台高效运行，政府法律顾问制度全面建立，法治政府建设不断加强。党风廉政建设和反腐败斗争深入推进，“庸懒散”治理全面加强，一批腐败分子得到依法惩处。刚刚过去的2015年，是“十二五”收官之年，也是我们迎难而上、攻坚突破的一年。这一年，世界经济复苏艰难曲折，国内经济下行压力不断加大，企业经营十分困难，特别是原油价格持续低迷，欧美对我国橡胶轮胎和石油管材进行“双反”调查，使东营经济发展面临“雪上加霜”的严峻形势。我们积极应对下行压力，科学谋划，迎难而上，经济保持平稳健康发展，生产总值增长6.9%，公共财政预算收入增长6.7%，全社会固定资产投资增长13.9%。化解了一批企业资金风险问题，全市贷款余额比年初增加431亿元、居全省第3位，增长16.6%、居全省第1位，不良贷款率全省最低。工业四大产业集群进一步壮大，化工园区和化工企业“三评级一评价”工作全面展开。水气污染整治专项行动持续深化，省控河流全部达到地表水五类水质标准，“蓝繁”天数增加28天、居全省第6位。中心城建设管理体制机制进一步理顺，东一路景观改造工程、西五路主体工程完工，植物园对外开放，拆违治乱和建筑市场打霸治痞两大专项行动强力开展。胜利机场国产大飞机试飞基地一期工程完工，德大铁路通车运营，东营火车南站投用，大唐东营电厂开工建设。向上争取支持取得重大突破，《环渤海地区合作发展纲要》把我市确定为重要节点城市，国务院批准设立黄河三角洲农业高新技术产业示范区、东营综合保税区，6家企业获得使用进口原油资质和配额1758万吨，油田生产建设用地城镇土地使用税恢复征收，京沪高铁第二通道列入国家中长期高速铁路网规划。这些都将对东营未来发展产生重大的推动和支撑作用。

　　各位代表，“十二五”的五年，是我市经济总量提升最快、城乡面貌变化最大、人民群众得实惠最多的时期之一。“十二五”的成就来之不易，这是在中央、省委省政府和市委正确领导下，全市上下、油地军校、社会各界团结奋斗、携手共建的结果。在此，我代表市政府，向付出辛勤劳动的广大干部群众，向积极支持政府工作的人大代表、政协委员和社会各界人士，表示崇高的敬意和衷心的感谢！

　　在看到成绩的同时，我们也清醒地认识到发展中还面临不少困难和问题。经济下行压力仍然较大，原油价格持续低位徘徊，部分企业生产经营困难，财政收支矛盾非常突出，就业压力进一步加大。农业基础仍然薄弱，工业转型升级进展不快，服务业发展相对滞后。水气污染整治任务十分繁重，安全生产还存在薄弱环节。社会民生领域仍有一些短板，经济社会发展不协调、城乡发展不平衡的问题依然突出。有的政府部门工作落实不力、服务效率不高、为政不勤不廉的现象仍然存在。我们必须采取有力有效措施，切实解决这些问题。

　　二、“十三五”经济社会发展的主要目标和任务

　　根据党的十八届五中全会和市委五届八次全会精神，市政府编制了《东营市国民经济和社会发展第十三个五年规划纲要(草案)》，已印发大会，请一并审议。

　　“十三五”时期，是东营资源城市转型攻坚期、战略地位提升跨越期、全面建成小康社会决胜期。我们要全面贯彻党的十八大和十八届三中、四中、五中全会精神，深入贯彻习近平总书记系列重要讲话精神，按照“四个全面”战略布局，坚持发展第一要务，以创新、协调、绿色、开放、共享五大发展理念为引领，以提高发展质量效益为中心，加快形成引领经济发展新常态的体制机制和发展方式，保持战略定力，坚持稳中求进，持续实施黄蓝国家战略，统筹推进经济建设、政治建设、文化建设、社会建设、生态文明建设，率先全面建成小康社会，向基本实现现代化阔步迈进，加快建设美丽幸福新东营。

　　“十三五”时期，我们要在提高发展平衡性、包容性、可持续性的基础上，保持经济7%左右的中高速增长，在全省率先实现经济总量和城乡居民人均收入比2010年翻一番，努力使转型发展步伐明显加快、人民生活水平明显提高、生态环境质量明显改善、体制机制活力明显增强、社会文明程度明显提升，把东营打造成为全国重要的高效生态经济示范区、资源型城市可持续发展引领区、环渤海地区重要节点城市和区域中心城市。

　　实现上述目标，要把握好五个方面的重点：

　　——牢固树立创新发展理念，着力推动经济转型升级。深入实施创新驱动发展战略，完善区域创新体系，强化企业主体地位和主导作用，实施一批重大科技项目，全社会研发经费占比提高到2.78%。发挥国家级农高区示范带动作用，实施农业提质增效转型升级行动，加快发展现代农业，在全省率先实现农业现代化。深入推进工业强市战略，实施创新驱动、信息化带动、公司规范化行动和严控准入“3+1”行动计划，推动传统产业高端发展、新兴产业规模发展、主导产业集群发展，打造东营制造业升级版，高新技术产业比重达到37%以上。突破发展现代服务业，打造黄河三角洲区域性服务中心，服务业比重达到38%左右。实施“互联网+”行动，推进信息化与产业深度融合。深入开展全国质量强市示范城市创建活动，构筑质量发展新优势。积极推进经济体制、行政管理体制等改革，形成有利于创新发展的体制机制和市场环境。

　　——牢固树立协调发展理念，着力构建平衡发展格局。推动城乡一体发展，协同推进新型城镇化、县域经济和新农村建设，有序推进外来务工人员市民化、城中村和城边村原有居民市民化、其他农村地区就地转移就业人口市民化，户籍和常住人口城镇化率分别达到61%和69%。推动区域融合发展，深化油地军校合作，完善共建机制，创新合作模式，形成多层次、宽领域、高效益的区域融合发展格局。推动物质文明精神文明协调发展，以实现“中国梦”为奋斗目标，积极倡导和践行社会主义核心价值观，统筹文化事业和文化产业改革发展，创建成为国家公共文化服务体系示范区。加快构筑立体交通体系，推进高速铁路、城际铁路、高速公路、机场、港口等重大工程建设，打开横贯东西、纵贯南北的通道；统筹推进水利、能源、信息等基础设施建设，全面增强综合支撑能力。

　　——牢固树立绿色发展理念，着力打造生态宜居城市。坚持生态立市、绿色惠民，推动形成绿色发展产业结构、生产方式、生活方式，争创国家生态文明示范区。实行最严格的环境保护制度，深化水气污染整治行动，加强土壤环境和农村环境保护，持续增加“蓝繁”天数。加强湿地保护与修复，开展大规模国土绿化行动，统筹推进海洋开发与生态保护，筑牢生态安全屏障，林木覆盖率达到21%。积极发展低碳循环经济，全面节约和高效利用资源。

　　——牢固树立开放发展理念，着力拓展发展空间。实施更加积极主动的开放战略，主动对接国家重大区域发展战略，全面融入对外开放新格局，以新一轮大开放促进新一轮大发展。实施以质取胜战略，优化市场布局和贸易结构，推动对外贸易向优进优出转变。坚持引进来和走出去并重，引资和引技引智并举，在更高层次上全面扩大对外开放。

　　——牢固树立共享发展理念，着力增进民生福祉。坚持居民收入增长和经济增长同步、劳动报酬提高和劳动生产率提高同步，持续增加居民收入。坚持就业优先战略，创造更多就业岗位，实现更加充分、更高质量的就业，城镇年均新增就业3万人。实施全民参保登记计划，加强制度衔接，形成覆盖全民、整合城乡、均衡协调的社会保障体系。坚持教育优先发展，大力发展各级各类教育，保障教育公平，提高教育教学质量，在全省率先实现教育现代化。统筹推进卫生、体育、养老、计生、残疾人等事业发展，加强食品药品安全监管，提高人民健康水平，建设健康东营。坚决打赢精准扶贫、精准脱贫攻坚战，2017年全面完成脱贫任务，确保全市人民同步奔小康。

　　三、2016年政府工作总体安排

　　今年是实施“十三五”规划的第一年，做好今年的工作，至关重要。我们要积极适应和引领经济新常态，全面落实宏观政策要稳、产业政策要准、微观政策要活、改革政策要实、社会政策要托底的总体思路，实施去产能、去库存、去杠杆、降成本、补短板五大任务，加快供给侧结构性改革和体制机制创新，努力提高经济发展质量和效益。主要预期目标是：生产总值增长7.5%左右，全社会固定资产投资增长12.5%左右，公共财政预算收入增长6.5%左右，完成省下达的节能减排约束性指标。重点做好以下十个方面的工作：

　　(一)努力保持经济稳定增长

　　持续扩大有效投资。充分发挥有效投资对稳增长调结构的关键作用，坚定不移实施项目带动战略，以优质增量拉动经济增长、带动存量调整，市级着力抓好77个重点建设项目、9个重点推进项目和140个政府投资项目，各县区、市属开发区分别突破一批重大项目。加大招商引资力度，加强与世界500强和行业领军企业的合作，鼓励企业与国内外知名企业进行资本、品牌、技术和渠道对接，运用好跨国并购、融资租赁等新的招商方式，吸引更多的跨国企业到东营投资落户。各级各部门要全力服务项目建设，加强要素资源保障，畅通“绿色通道”，实行全程跟踪服务和专项督查考核，保障重点项目顺利落地开工。创新投融资机制，推广PPP模式，面向全社会推出一批合作项目。

　　保障企业稳定发展。稳增长关键是稳企业。引导企业顺应供给侧结构性改革趋势，减少无效和低端供给，扩大有效和中高端供给，在满足一般消费需求的同时，多生产绿色、健康、智能的高附加值产品。引导企业多措并举消化库存，帮助企业降低制度性交易成本、税费负担、社会保险费、财务成本、物流成本。加强政银企合作，引导金融机构增加信贷投放，帮助企业化解融资难问题。加快规范化公司制改制，鼓励企业采用上市挂牌、债券融资等方式，拓宽直接融资渠道，力争年内10家企业上市或挂牌。支持企业主导或参与制定行业标准、国家标准和国际标准，提升研发设计和品牌创建能力。稳妥化解过剩产能，对丧失自我修复能力的企业，通过兼并重组、债务重组、破产清算等方式，实现市场出清。

　　企业家是东营发展的宝贵财富。大力实施优秀企业家培养工程，组织企业家到国内外名企名校集中学习，邀请知名经济学家开展培训，造就一大批优秀企业家和高素质企业经营管理人才。要尊重、关心、保护企业家，让企业家政治上有地位、经济上有回报、生活上有关照。广大企业家要大力弘扬创新精神和工匠精神，心怀梦想、志存高远，脚踏实地、精益求精，对标国内外一流企业，扬长补短，拼搏创业，倾力打造百年企业。

　　(二)以创新引领工业转型升级

　　坚持工业强市战略不动摇，以提质增效、转型升级为主攻方向，加快发展先进制造业，推动由工业大市向工业强市转变。四大产业集群是东营工业发展的重要基础，要加快应用新技术、新装备、新工艺、新材料，提高产业层次，推动创新发展和内涵发展。橡胶轮胎及汽车配件产业，重点在提高产业集中度、提升品牌影响力上下功夫，支持企业以资产和品牌为纽带，进行重组、兼并，努力向高端化迈进。有色金属产业，重点是向精深加工方向发展，依托现有骨干企业，加快产业链延伸，开发一批高精尖产品，努力提高产品的附加值。石油装备产业，重点是加强与国际石油装备巨头的合资合作，搞好国内外研发中心、生产基地和营销网络建设，鼓励企业以工程总承包形式带动装备、设计、施工全产业链“走出去”，尽快做大做强。石油化工及盐化工产业，抓住我市企业获批原油进口和加工贸易成品油出口资质的机遇，加快推进产业布局基地化、新上项目规模化，制定完善配套政策，推动企业向东营港经济开发区等北部片区聚集，打造国家级临港石化产业基地。打好化工产业转型升级攻坚战，严格开展“三评级一评价”，发展壮大一批、规范提升一批、关闭淘汰一批。支持四大产业集群分别建立一批高水平的创新平台、公共检测平台、产品展销平台和物流平台，努力把四大产业集群做大做强。

　　新兴产业科技含量高、环境污染小，对工业转型升级具有重大引领带动作用。要加大对新兴产业的扶持力度，充分发挥政府产业投资引导基金作用，鼓励企业选择有基础有条件的领域进行集中突破，积极发展新能源、节能环保、新一代信息技术、新材料、现代生物与医药等产业，尽快形成新的发展支撑。加快国产大飞机试飞基地建设，积极发展航空产业。

　　强化企业创新主体地位，鼓励大企业加强自主创新能力建设，借助国内外知名高校企业和科研院所的力量，跟踪国际新技术发展，突破关键核心技术，培育一批创新型领军企业；支持中小企业深化产学研结合，朝着“专、精、特、新”方向发展。打造可持续发展研究院、现代农业研究院等重大研发平台，积极创建电子陶瓷材料等国家级创新平台。支持胜利经济开发区创建石油装备产业国家高新区、广饶经济开发区创建国家级开发区。推进科技金融结合，设立中小企业贷款风险资金池，建设科技金融企业库，组建科技金融服务中心，规划建设创新广场。深入实施重大人才工程，完善政策措施，大力引进高水平创新创业团队，培养引进科技领军人才和急需紧缺的专业技术人才，加强与国家“千人计划”专家及团队的合作。推进国家科技服务试点城市和国家知识产权示范城市建设。

　　(三)大力发展现代农业

　　提升优势产业发展水平。畜牧业，坚持生态化发展方向，提高产业竞争力和畜产品附加值，加快现代畜牧业示范区发展。渔业，以现代渔业园区为重点，加强苗种改良和养殖新技术推广，进一步提高产品规格质量，推进“海上粮仓”建设。食用菌产业，以工厂化食用菌为主要发展方向，重点搞好高档珍稀食用菌品种研发和先进适用技术推广，全面提升核心竞争力。种植业，实施“渤海粮仓”科技示范和粮食高产创建工程，巩固提升粮食生产能力；积极发展高效经济作物，开展粮改饲试点；着力培育优质特色林果基地和种苗花卉产业，大力发展林下经济。支持国家农高区创新发展，打造“农业硅谷”，发挥示范引领作用。积极发展“第六产业”，推动一二三产业深度融合。

　　深化提高农业产业化经营。实施农业龙头企业集群发展战略，加大政策扶持力度，抓好高端乳制品、进口肉牛加工等农产品精深加工项目，建设一批高标准生产基地，提升农业规模化、标准化、品牌化发展水平。大力发展休闲观光农业，打造一批各具特色的农业综合体。积极发展电子商务等新型流通业态，完善农产品营销网络，开拓中高端销售市场。开展农民专业合作社和家庭农场示范创建活动。

　　加强农田水利建设。加快南水北调配套工程建设。对溢洪河、支脉河、马新河等骨干河道进行综合治理。实施王庄、麻湾、曹店等5个灌区配套改造。抓好六大片区26.9万亩田间水利配套工程建设。拓宽农田水利建设融资渠道，创新管养机制。实施三年增绿计划，今年造林绿化13.3万亩，修复保护湿地10万亩。

　　推进农村综合改革。有序推进农村集体土地承包经营权、林权、水域滩涂养殖使用权和农村承包土地经营权确权颁证工作，加快县级农村产权交易中心建设，扎实推进新型农村合作金融试点、供销社综合改革试点，加快黄河三角洲土地金融控股公司筹备组建。发挥新型农民学校作用，大力培养新型职业农民。完成省定标准贫困人口脱贫。

　　(四)突破发展现代服务业

　　坚持生产性服务业和生活性服务业一起抓，壮大规模、提升档次，提高服务业供给质量。积极发展金融业，做大做强地方法人金融机构，支持银行业金融机构拓展业务空间，创新金融产品和服务，大力发展现代保险和证券业，实现金融与实体经济互促互进、融合发展；继续大力发展私募市场和股权投资基金，探索发展股权众筹、第三方支付等互联网金融，稳妥推进介于现货与期货之间的大宗商品交易试点，探索建立金融控股、资产管理公司；着力去杠杆，规范信托、理财等表外融资业务，降低金融资产风险；加强全方位金融监管，积极化解企业间担保链、担保圈风险，严厉打击非法金融活动，坚决守住不发生系统性区域性风险底线，维护良好金融秩序。大力发展现代物流业，依托港口、机场、铁路站场等交通枢纽，规划建设一批物流园区；依托主导产业，培育发展一批物流企业；支持快递业规范提升、加快发展。加快推进黄河口生态旅游区创建国家5A级景区工作，支持重点旅游景区开展国家A级景区创建升级活动，打造一批乡村旅游亮点。编制养老服务设施建设规划，加强新型养老社区建设，推进医养结合，鼓励社会资本发展健康养老产业。积极发展人才和家政服务业。把发展服务业与扩大消费有效结合起来，加强消费者权益保护，积极扩大消费需求。

　　(五)继续深化“三大专项行动”

　　城市公共安全专项行动。深化安全生产网格化管理，落实企业安全生产主体责任，层层传导工作压力，健全完善公共安全责任体系。加快安全生产数字化监管平台建设，提升安全监管执法能力，推动企业安全生产管理工作标准化、各级安全生产管理信息化。围绕改善道路交通通行环境、增强消防防火灭火能力、推动“科技兴安”等方面，组织实施一批重点项目。深入推进安全生产隐患大排查、快整治、严执法集中行动，预防和减少各类生产安全事故。

　　水气污染整治专项行动。以环境质量改善为核心，突出抓好污水管网配套、人工湿地水质净化工程和污水处理厂建设运营，积极推进城区雨污分流改造，全面消除污水直排口，市控以上河流稳定达到地表水五类标准。严格执行大气污染物排放标准第二时段限值，突出抓好燃煤电厂、10吨以上燃煤锅炉超低排放改造，加快淘汰10吨以下燃煤锅炉，今年中心城关停胶州路、滨州路供热站等燃煤锅炉，加强石油化工等行业挥发性有机物整治和扬尘污染治理，环境空气质量改善10%以上。严格能耗和污染排放源头控制，大力发展循环经济。严格执行新环保法，重拳打击环境违法行为。

　　乡村文明建设专项行动。按照农村环境建设标准，规范有序推进农村环境治理，年内典型示范村占比达到45%、环境治理村全面转化提升。持续推进农村清脏治乱，深化环卫一体化工作，年内完成120个村庄的污水治理工作，用三年时间完成农村旱厕改造任务。

　　(六)加强城市和重大基础设施建设

　　加强中心城规划建设管理。以争创联合国人居环境奖为目标，开展“城市生态环境建设年”活动，全面提升中心城规划建设管理水平。坚持“大空间、大绿地、大水面、大湿地”的城市特色，高水平编制各类规划，积极推进“多规合一”，严格执行规划编制审批和修改程序，三年内实现控制性详规全覆盖。强化城市设计，重点抓好中心城总体设计、东城核心区、西城核心区等重点片区城市设计，制定实施城市设计管理办法和技术导则。加强城市道路建设改造，开工建设南一路快速路、西一路北延、德州路，综合改造南二路、东二路、西一路、西二路。提升景观亮化水平，实施府前大街、东二路等景观亮化提升工程。完善市政设施，加快推进广北水库水源地和配套管线建设，加强背街小巷整治，启动中心城区地下综合管廊规划建设，研究制定海绵城市建设实施方案。加强城市生态建设，规划建设中心城森林湿地公园、广利河生态廊道，建设绿道104公里、街头游园15处，对清风湖、秋月湖、锦华雕塑、龙熙新都等8个城市公园进行景观提升，对利三沟水系进行高水平改造，打造成市区带状公园。巩固文明城市创建成果，提升城市精细化管理水平。充分发挥市场竞争机制，建立对管护队伍的考核奖惩制度，提高城市管护成效。加强“智慧东营”建设，年内建成“市县统一、多级应用”的政务云计算中心。强势强力开展拆违治乱、打霸治痞专项行动，力争一至两年内中心城、县城、建制镇实现建成区内无违建的目标。落实公积金使用、信贷、税收等政策，打通保障房和商品房通道，积极消化房地产库存。

　　加强县城、小城镇、社区规划建设。深入开展国家和省市试点示范县、示范镇创建，加快东营港城建设，加强棚户区、城中村、城边村和危房改造，推进城镇一体化管理。三年内完成市区和县城棚户区、老旧住宅小区、城中村改造。加强城镇绿化，每个县区打造2个绿化示范镇和20个左右的绿化示范村，创建一批绿色村庄。以网格化、信息化为手段，提升城市社区规范化建设水平。实施农村社区服务中心建设三年行动计划，支持符合条件的农村社区纳入城镇体系管理。继续抓好黄河南展区新型农村社区建设。

　　加强重大基础设施建设。启动济南至东营高铁项目前期工作，争取京沪高铁第二通道列入国家“十三五”重点建设项目，争取利用德龙烟铁路复线建设山东北部快速铁路通道。加快推进黄大铁路建设，全面实施淄东铁路扩能改造工程。争取东营港疏港铁路等项目尽快落地实施。济东高速公路年底前通车。加快长深高速广饶至高青段建设，争取继续向西延伸到济南机场，列入省“十三五”规划尽快实施。推动环渤海高等级公路尽快启动建设。完成《东营港总体规划》修编，加快三突堤码头及罐区建设，推进10万吨级深水航道和南防波堤等重大工程立项、建设，开展10-30万吨油轮过驳业务。加快广利港航道防波堤、通用码头、广利河防洪北堤工程建设，明年实现开港通航。继续增开优化胜利机场航线航班，深化与中国商飞公司合作，实施跑道延长工程，满足大飞机试飞。加快500千伏垦东输变电工程、大唐东营电厂建设，开展胜利电厂第二台机组前期工作。

　　(七)加快区域一体化发展

　　深度融入区域大发展格局。主动对接环渤海、京津冀区域发展战略，全力承接北京非首都功能疏解和京津产业转移。积极对接“一带一路”战略，参与中韩、中澳自贸区建设。加强与黄河经济带、长江经济带各城市的经济交流和技术合作，实现优势互补、共赢发展。

　　推动油地军校合作共建向深向实发展。创造良好环境，支持胜利油田积极应对低油价，深化改革创新，加快转型发展；发挥胜利油田、中国石油大学科技、人才优势，促进区域产业升级和城市转型。围绕城市和基础设施、科技人才合作、安全环保、社会民生等领域，实施一批新的油地校共建项目。支持中国石油大学东营校区建设，积极推进油地校共建中国石油大学胜利学院，努力办成区域内一流高校。大力支持驻地部队建设，打造军地军民融合发展的典范。

　　积极发展县域经济。支持各县区发挥各自优势，明确功能定位，打造各具特色的经济板块。统筹推进基础设施、产业布局、环境保护、社会民生，提高一体化发展水平。继续实行促强扶弱政策，支持强县争先进位，支持利津县加快科学发展。

　　(八)持续深化改革开放

　　深化重点领域和关键环节改革。深化商事制度改革，加强事中事后监管，启动公共信用信息平台建设。深化预算编制等改革，全面推行预决算公开；依法加强税收、非税收入征管和财政专项资金整合监管，严格控制一般性支出；完善政府债务管理机制，防范化解债务风险。深化国有企业改革，健全国有资产管理体制机制。加快推动未利用地开发，建设土地整治部级实验基地，开展不动产统一登记。深化医药卫生体制改革，全面推进全国公立医院综合改革试点，探索建立符合行业特点的人事薪酬制度和分级诊疗的有效实现形式，不断完善基层医疗机构运行机制。深化教育领域综合改革，实施小班化教学、集团化办学，改革完善初中学生学业考试及高中阶段学校招生制度，推进校长教师交流轮岗，试行合同制教师制度改革。深化行政管理体制改革，加快事业单位分类改革。

　　积极扩大对外开放。实施优进优出战略，培育壮大以技术、品牌、质量、服务为核心的对外经济新优势。大力开拓国际市场，实施境外百展计划，鼓励企业开展跨境电子商务，支持企业在海外建立国际营销和售后服务网络，主动应对国际贸易摩擦。提升石油装备、橡胶轮胎、精细化工出口基地建设水平，提高出口产品的科技含量和附加值。扩大原油进口，积极争取开展原油加工贸易；支持企业有选择地进口能源资源性产品、关键设备和重要零部件。加强电子口岸和大通关建设，推动东营港码头对外开放。加快东营综合保税区建设，确保6月份封关运营；加紧招商引资，尽快落地一批重大项目，发挥引领开放型经济发展的作用。办好橡胶轮胎展、石油装备展、石油化工展等高端展会，不断提高水平、增强效果，促进我市主导产业国际化发展。引导企业扩大对外交流，支持企业走出去，开展国际产能合作。

　　(九)着力保障改善民生

　　积极推动就业创业。实施更加积极的就业政策，突出抓好淘汰落后产能、企业转型升级过程中职工分流安置及失业人员再就业工作，统筹高校毕业生、农村富余劳动力、就业困难人员等群体就业。坚持以创业带动就业，完善创业就业服务体系，建立面向人人的服务平台，鼓励大众创业、万众创新。规范劳动用工管理，推行农民工实名制管理制度，建立和谐劳动关系。

　　提高社会保障水平。继续提高城乡低保、农村五保供养标准。开展全民参保登记，基本实现法定人员全覆盖，继续做好政府购买养老服务等工作。完善职工养老保险金个人账户制度。健全医疗保险稳定可持续筹资和报销比例调整机制，完善按病种分值结算为主的付费方式。完善城乡居民大病保险制度，建立职工大病保险制度，开展居民长期护理保险制度建设前期工作。落实被征地农民社会保障政策。统筹城乡救助体系，加快推进“大救助”平台建设，支持发展慈善和社会福利事业。深入实施残疾人“整体赶平均、共同奔小康”行动。

　　坚持教育优先发展。落实教育发展保障机制，深入推进第二期学前教育三年行动计划，扶持普惠性民办幼儿园，扩大普惠性学前教育资源。推进义务教育优质均衡发展，利津县年内通过义务教育发展基本均衡县国家认定。抓好城乡学校、幼儿园建设，实施解决“大班额”问题专项规划，到2017年全市新建、改扩建63所中小学。加快市中专、特校等学校建设，市一中、二中新校秋季入学启用。加快发展现代职业教育，推进产教融合、校企合作，着力培养高技能人才。支持和规范民办教育发展。

　　提高人民健康水平。建设便民惠民、互联互通、资源共享的人口健康信息化系统。提升基层医疗卫生服务能力，建立乡村医生定向培养制度。加快推进公共卫生服务机构建设，推动公共卫生服务均等化。支持社会力量办医，鼓励发展中医药事业。积极申报建设住院医师规范化培训基地。深入开展爱国卫生运动，做好国家卫生城复审工作。落实“二孩”生育政策，提高出生人口素质，促进人口均衡发展。

　　推动文化繁荣发展。坚持用“中国梦”和社会主义核心价值观凝聚共识，汇聚力量，加强思想道德建设和社会诚信建设，培育良好社会风尚。扎实推进国家公共文化服务体系示范区创建工作，实施文化惠民工程，完善公共文化服务体系。支持发展骨干文化企业和创意文化产业，积极培育新兴文化业态。发展积极向上的网络文化。弘扬传承优秀传统文化，实施历史文化展示和乡村记忆工程，支持孙子文化、吕剧等地方文化精品的研究和发展。加强各级档案馆建设。倡导全民阅读。实施全民健身工程，大力发展体育事业和体育产业。

　　加强食品药品安全监管。全面推进监管制度改革创新，加强三级监管队伍和技术装备建设，提升食品药品安全治理能力。健全农产品、食品、药品等重要产品追溯体系，深入推进示范创建和专项整治，加强市场监管执法，提高产品质量安全水平。扎实推进出口农产品质量安全示范市和农产品质量安全县创建工作。

　　创新社会治理。加强法制宣传教育，启动“七五”普法依法治理工作，开展多形式法治创建活动，增强全民法治观念。进一步加强人民调解、社区矫正和安置帮教工作，推进公共法律服务体系建设。加强和改进信访工作，健全矛盾纠纷排查化解机制，及时就地解决群众合理诉求。深入推进平安东营建设，完善立体化社会治安防控体系，加强油区安全综合整治，依法严密防范和惩治违法犯罪活动，确保社会秩序持续稳定。

　　办好民生实事。(1)建设城市绿道和街头游园。(2)启动建设新型养老社区。(3)建设就业创业基地，培训创业创新人员。(4)实施农村公路安全生命防护工程。(5)更新新能源公交车，优化公交线路。(6)改造提升中心城老旧住宅小区40个。(7)实现天然气镇镇通。(8)建设100个乡村剧场、100个群众文化广场。(9)扩大公共自行车试点，增设自行车站点140个。(10)启动实施农村旱厕改造。群众利益无小事。我们一定坚持民生为先、民生为重、民生为本，把惠及民生的事情做实做细做好，让人民群众在共建共享发展中有更多获得感！

　　(十)加强政府自身建设

　　做好今年工作，完成各项目标任务，必须全面加强政府自身建设。我们要建设阳光政府。持续推进限权放权、放管结合、优化服务，不断提高办事效率和服务水平。深化政务公开，不断增强政府透明度和公信力。我们要建设法治政府。深入推进依法行政，把政府活动全部纳入法治轨道。坚持依法科学民主决策，凡重大决策必须严格履行法定程序。改进和加强行政执法，推进综合执法，做到严格规范公正文明执法。认真执行市人大及其常委会决议、决定，做好人大代表建议和政协提案办理工作，自觉接受各方面监督。我们要建设服务政府。牢固树立服务观念，巩固和拓展党的群众路线教育实践活动和“三严三实”专题教育成果，驰而不息改进作风。着力增强看齐意识、担当意识、标准意识、效率意识，力戒“不作为、慢作为”现象，心无旁骛干工作，只争朝夕抓发展，锲而不舍抓落实。我们要建设清廉政府。坚持从严治政，严守政治纪律和政治规矩，进一步加强惩治和预防腐败体系建设，把权力关进制度的笼子。坚持无禁区全覆盖零容忍，坚决惩处各类违法违纪行为。

　　各位代表！实现新发展任务艰巨，创造新业绩使命光荣。让我们紧密团结在以习近平同志为总书记的党中央周围，在省委、省政府和市委的坚强领导下，坚定信心、改革创新，攻坚克难、扎实工作，为加快建设美丽幸福新东营而努力奋斗！
